# Genome Sequences of a Novel HIV-1 Circulating Recombinant Form (CRF61_BC) Identified among Heterosexuals in China

**DOI:** 10.1128/genomeA.00326-13

**Published:** 2013-06-27

**Authors:** Xingguang Li, Chuanyi Ning, Xiang He, Yao Yang, Fan Li, Hui Xing, Kunxue Hong, Rongge Yang, Yiming Shao

**Affiliations:** State Key Laboratory for Infectious Disease Prevention and Control, National Center for AIDS/STD Control and Prevention, Chinese Centre for Disease Control and Prevention, Beijing, Chinaa; HIV Molecular Epidemiology and Virology Research Group, the State Key Laboratory of Virology, Wuhan Institute of Virology, University of Chinese Academy of Sciences, Wuhan, Hubei, Chinab

## Abstract

We report here the first novel HIV-1 second-generation intercirculating recombinant form 61_BC (inter-CRF61_BC), composed of two established CRFs (CRF07_BC and CRF08_BC), in China. CRF61_BC is the first CRF found among the heterosexual population in two different regions in China, suggesting the increasing significance of heterosexual transmission of HIV in China.

## GENOME ANNOUNCEMENT

Human immunodeficiency virus type 1 (HIV-1) exhibits an extensive genetic diversity that is primarily driven by high rates of mutation, recombination, and replication. HIV-1 group M strains play a major role in the global HIV-1 epidemic and are further classified into eleven subtypes and sub-subtypes (A1, A2, B, C, D, F1, F2, G, H, J, and K), as well as a number of circulating recombinant forms (CRFs) with various unique recombinant forms (URFs). To date, 58 CRFs have been reported in the Los Alamos National Laboratory HIV database (http://www.hiv.lanl.gov/content/sequence/HIV/CRFs/CRFs.html). Up to now, three CRFs have been reported for the first time in China: CRF07_BC (1), CRF08_BC (2), and CRF57_BC (3). Our national cross-sectional study of HIV molecular epidemiology showed that CRF07_BC and CRF08_BC were the main predominant strains, accounting for 55.6% of reported infections, and were mainly epidemic among injecting drug user (IDU) and heterosexual populations in China in 2006 (4). Cocirculation and dual infections of CRF07_BC and CRF08_BC among IDU and heterosexual populations in China provide the opportunities to generate various types of new URFs and CRFs. In this study, we report the first novel HIV-1 second-generation intercirculating recombinant form (inter-CRF), designated CRF61_BC, composed of two established CRFs (CRF07_BC and CRF08_BC), which were identified in the heterosexual population in two different regions in China.

Blood plasma samples were collected from three HIV-positive consenting subjects (strains 07CN.JL070009, 10CN.JL100010, and 07CN.FJ070004) who were infected through heterosexual transmission in Jilin and Fujian. The study was approved by the institutional review board of the National Center for AIDS/STD Control and Prevention. Near-full-length-genome (NFLG) sequences (>9.0 kb) were determined from plasma HIV-1 viral RNA by single-genome amplification methods with primers that have been described previously (5). Amplicons were purified using the QIAquick gel extraction kit (Qiagen) and sequenced directly by an ABI 3730XL sequencer using BigDye Terminator (Applied Biosystems) by Beijing (China). All sequenced data were cleaned and assembled into contiguous sequences using the Sequencher program version 5.1 (Gene Codes Corporation). The codon-aligned nucleotide sequence alignment was constructed using the Gene Cutter online tool, available from the Los Alamos National Laboratory HIV Sequence Database (http://www.hiv.lanl.gov/content/sequence/GENE_CUTTER/cutter.html), and then minor manual adjustments according to codon-aligned nucleotide sequence alignment were made using BioEdit v7.0.9 (6). Phylogenetic analyses were performed using MEGA v5.05 (7). Recombination analyses were performed using jpHMM, RIP, and SimPot v.3.5.1 (8). The NFLG sequences from the three subjects were 9,087, 9,080, and 9,024 bp in size for strains 07CN.FJ070004, 07CN.JL070009, and 10CN.JL100010, respectively. These three strains formed a distinct monophyletic cluster between the CRF07_BC and CRF08_BC clusters and are distantly related to all known HIV-1 subtypes or CRFs. Recombinant analyses revealed that these three strains were composed of CRF07_BC and CRF08_BC. This recombinant structure is distinct from any known CRFs reported to date. Therefore, these new recombinants are now designated CRF61_BC. CRF61_BC comprises the fourth CRF identified in China. Other examples known to date are CRF07_BC, CRF08_BC, and CRF57_BC. The emergence of this CRF suggests the presence of active transmission networks of HIV infections among the heterosexual population in China.

### Nucleotide sequence accession numbers. 

The sequences are available in GenBank under the accession no. KC990124 to KC990126.
